# Gastric Pouch Resizing for Recurrent Weight Gain After Roux-en-Y Gastric Bypass—Does It Have Its Rational?

**DOI:** 10.1007/s11695-024-07581-y

**Published:** 2024-11-12

**Authors:** Stefanie Josefine Hehl, Dominique Lisa Birrer, Renward Hauser, Daniel Gero, Andreas Thalheimer, Marco Bueter, Jeannette Widmer

**Affiliations:** 1 Department of Visceral and Transplant Surgery, Swiss HPB Center, University Hospital Zurich, 8091 Zurich, Switzerland; 2 Department of Surgery, Männedorf Hospital, 8708 Männedorf, Switzerland; 3 Department of General, Visceral and Transplant Surgery, University Hospital Tübingen, Hoppe-Seyler-Strasse 3, 72076 Tübingen, Germany; 4 Faculty of Medicine, University of Zurich, 8091 Zurich, Switzerland

**Keywords:** Pouch resizing, Recurrent weight gain, Bariatric surgery

## Abstract

**Introduction:**

The most effective treatment for obesity and associated comorbidities is metabolic-bariatric surgery (MBS). Nevertheless, recurrent weight gain is reported in up to 40% of patients after Roux-en-Y gastric bypass (RYGB), eventually with a recurrence of obesity-associated comorbidities. Gastric pouch resizing (GPR) is performed as a low-risk secondary surgery to cease weight regain. We herewith analyzed the effect of GPR after primary RYGB on long-term weight loss, course of comorbidities, safety, and patient satisfaction.

**Methods:**

Forty-eight patients undergoing GPR between 2016 and 2020 at the University Hospital of Zurich were included. Data were collected from a prospective database. GPR was performed laparoscopically and included a resection of the enlarged gastric pouch and a redo of the gastrojejunostomy. Additionally, 37 patients participated in a survey to evaluate PROMs (patient-reported outcome measures).

**Results:**

GPR followed RYGB after a mean time of 106.2 ± 45.5 months at a mean BMI of 39 ± 5.4 kg/m^2^. Mean follow-up was 55.9 ± 18.5 months with a mean BMI 1- and 5-years postoperative of 37 ± 5.5 kg/m^2^ and 35 ± 7.5 kg/m^2^, respectively. Obesity-associated comorbidities were resolved in 53% of patients at follow-up (*p* < 0.05)**.** Minor postoperative complications occurred in 12.5% while major complications occurred in 10.4% of patients. The PROMs showed high levels of satisfaction after GPR.

**Conclusion:**

GPR for recurrent weight gain after primary RYGB is a safe procedure resulting in weight stabilization and resolution of obesity-associated comorbidities. It is thus a valuable surgical option in well-selected patients.

**Graphical Abstract:**

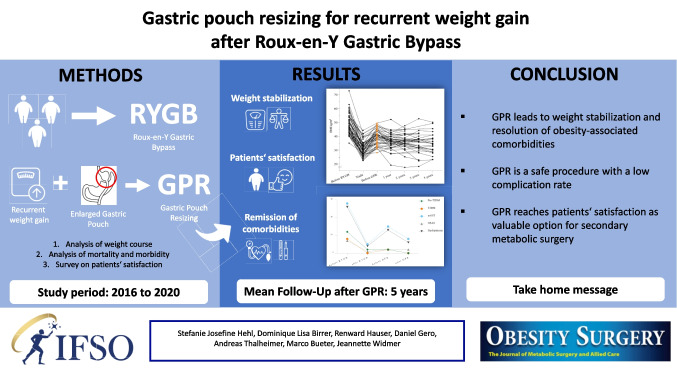

**Supplementary Information:**

The online version contains supplementary material available at 10.1007/s11695-024-07581-y.

## Introduction

The most effective and sustained treatment for severe obesity is nowadays metabolic-bariatric surgery (MBS)—with laparoscopic Roux-en-Y gastric bypass (RYGB) being one of the most frequently performed primary MBS [1]. This technique not only promotes optimal weight loss but also leads to a resolution of obesity-associated comorbidities [2–4]. Nevertheless, a significant recurrent weight gain may occur in up to 40% of patients after years and is often escorted by a relapse of comorbidities [5–7]. With an increasing prevalence of obesity and associated comorbidities worldwide, the need for MBS will be followed by a growing demand to address recurrent weight gain or even suboptimal weight loss. The reasons for recurrent weight gain are multifactorial, reaching from hormonal imbalances and dietary non-adherence to anatomical factors suggesting technical failure during primary procedure [8]. As complex and multilayered as the effect of MBS on weight reduction is, so are the reasons for recurrent weight gain. Accordingly, it is assumed that different, individualized therapies are needed to stop and/or reverse recurrent weight gain. The challenge is to find out which therapy will be best for whom. However, weight stabilization and a resolution of recurrent obesity–associated comorbidities should be counted as an optimal clinical response to secondary treatment.

Enlarged gastric pouch sizes (often due to unintentional fundus residuals), dilation of the gastrojejunal anastomosis, and/or delayed pouch emptying are factors that may contribute to a loss of satiety with subsequent recurrent weight gain [9]. GPR and a redo of the gastrojejunostomy intend to reestablish the pouch size, hence restoring efficient pouch emptying. In this study, we aim to understand the effects of GPR after RYGB on long-term weight loss, course of comorbidities, and safety by analyzing our data. Eventually, patients’ satisfaction was evaluated with a self-designed survey.

## Material and Methods

### Data Collection and Study Population

A prospectively collected database of patients who underwent GPR between January 2016 and December 2020 at the University Hospital of Zurich was retrospectively analyzed. Patients after primary RYGB with recurrent weight gain as defined below over the age of 18 years were included if GPR was chosen as secondary MBS. Patients who underwent a redo of the gastrojejunostomy with or without GPR for any other reasons (e.g., anastomotic ulcers, leakage, or bleeding) were excluded. Follow-up was performed at 3 months and 1, 2, 3, and 5 years after GPR. The collected data encompassed patient demographics, weight history, percentage total weight loss (%TWL) [10], obesity-associated comorbidities, operation time, length of hospital stay, and complications monitored with the Clavien-Dindo classification (CDC) [11] and Comprehensive Complication Index (CCI) [12]. The CCI, summarizing all postoperative complications ranging from 0 (no complication) to 100 (death), was calculated for hospital stay and at 90 days after surgery. At the end of the study period, patients were asked to participate in a PROM (patient-reported outcome measures) via phone call (Supplementary 1).

### Preoperative Management

All patients were discussed in our interdisciplinary obesity board before and after a thorough physical and psychological assessment. The final indication for GPR was met when patients presented with a regain of more than 20% from nadir-weight after RYGB along with or without a recurrence of comorbidities and with an enlarged gastric pouch. This regimen was approved by all specialties participating. All patients received counseling from dieticians and psychologists to enhance compliance. Preoperative workup consisted of an upper gastrointestinal endoscopy and X-ray series with the evaluation of gastric pouch size and emptying dynamics by radiologists or bariatric surgeons.

### Surgical Technique

The primary RYGB was performed by overall five bariatric surgeons over a period of 24 years. The technique for the gastrojejunostomy was changed in 2016 from circular- to linear-stapled anastomosis—not affecting the principles of the gastric pouch creation. The gastric pouch was built either by 60-mm or 45-mm linear staplers (depending on the surgeon’s preference), one horizontal and 2–3 vertical staples towards the angle of His.

GPR was performed laparoscopically by three experienced bariatric surgeons (each with > 50 MBS per year). First, all the adhesions around the gastric pouch were completely removed to assess the size of the gastric pouch and the gastrojejunostomy without restriction. With a horizontal resection above the gastrojejunostomy, the gastric pouch was detached from the jejunum. After inserting a large gastric tube, lateral resection towards the angle of His along the tube was performed to create a slim gastric pouch. The jejunum was mobilized in such a way that a tension-free anastomosis was possible. The gastrojejunostomy was performed with a circular (until 2016) or linear stapler (after 2016). The resected part of the gastric pouch as well as the primary gastrojejunostomy were excised from the jejunum close to the new anastomosis and were removed via an access port in an endo bag. Finally, a blue-dye test was performed to rule out leakage on the new gastrojejunostomy.

### Definitions

Recurrence and remission of obesity-associated comorbidities were defined as follows: Prediabetes was characterized by an HbA1c range of 5.7 to 6.4%. Diabetes, arterial hypertension, and obstructive sleep apnea syndrome (OSAS) were determined as the presence and discontinuation of antidiabetic/antihypertensive medications and/or CPAP (continuous positive airway pressure) therapy. Dyslipidemia was defined as any deviation of blood lipids from normal values and the return to normal values after GPR. Minor complications were defined as analog CDC ≤ II and major complications as analog CDC ≥ III.

### Statistical Analysis

Statistical significance was defined as *p* < 0.05. Continuous data are shown as mean ± standard deviation (SD) as appropriate and categorical as number (*n*) and percentage (%) in a descriptive manner. Continuous variables normally distributed were compared with the paired *t*-test. Differences in nominal data were compared by Fisher’s exact test. GraphPad Prism Version 9.1.0 (GraphPad Software, Inc., San Diego, CA) was used for statistics.

## Results

Within the study period, a total of 80 patients underwent GPR after primary RYGB. Thirty-two patients were excluded due to GPR for other reasons than recurrent weight gain. Out of the remaining 48 patients, 39 (81.3%) were female with a mean age of 46 ± 10 years at the time of GPR. The mean operation time was 117 ± 33 min (Table 1). The mean time after the primary RYGB until the GPR operation was 106 ± 45.5 months, with a minimum of 23 and a maximum of 229 months.

**Table 1:** Baseline characteristics

Parameter	Gastric pouch resizing
Overall *n*	48
Female *n*	39 (81.3)
Age (years)	46 ± 10
BMI baseline (kg/m^2^)	49 ± 7.3
BMI nadir (kg/m^2^)	31 ± 5.2
Time after RYGB (months)	26.3 ± 19.4
BMI before GPR (kg/m^2^)	39 ± 5.4
Time to GPR (years)	8.9 ± 3.8
Operation time (min)	117 ± 32.7
LOS (days)	5.1 ± 1
Follow-up post GPR (months)	55.9 ± 18.5

Values in (%) or mean (± SD)

*BMI* body mass index, *RYGB* Roux-en-Y gastric bypass, *GPR* pouch resizing, *LOS* length of hospital stay

### Preoperative Workup

An upper endoscopy was performed in 81.3% (*n* = 39) patients before GPR. An enlarged gastric pouch with a volume greater than 60 ml was documented in 64.1% (25 out of 39) patients, whereas a widening of the gastrojejunal anastomotic diameter greater than 2 cm was observed in 5.1% (2 out of 39) patients.

**Fig. 1:**
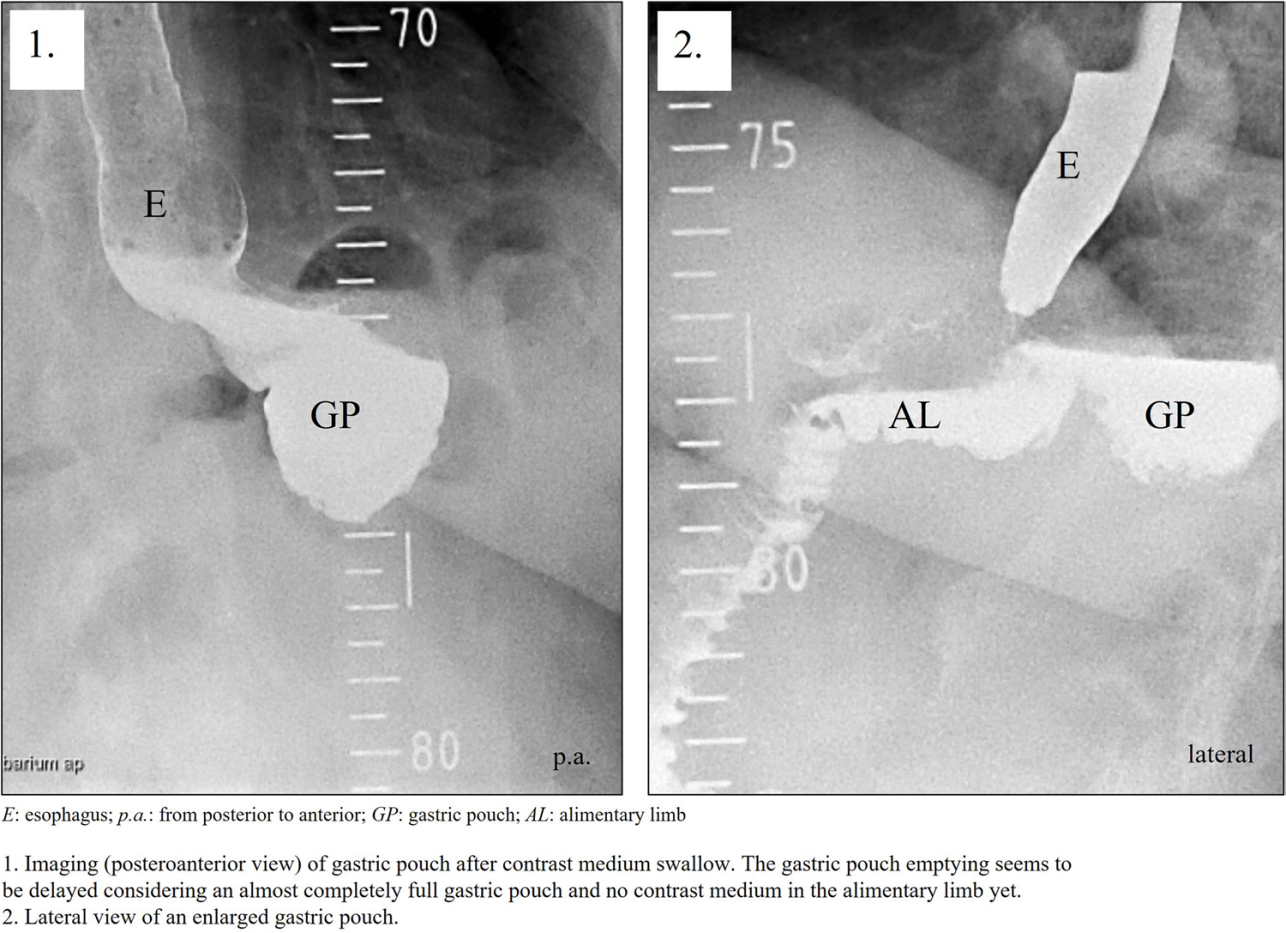
Contrast medium swallow X-ray

A total of 95.8% (*n* = 46) patients received contrast imaging before GPR. An enlarged gastric pouch was documented in 87% (40 out of 46) on contrast imaging, with delayed gastric pouch emptying recorded in 67.4% (31 out of 46) patients (Fig. 1).

Overall, a total of 22 (45.8%) patients had recurrent weight gain alone and an enlarged gastric pouch. Eight (16.7%) patients exhibited both recurrent weight gain and recurrence of one or more comorbidities along with an enlarged gastric pouch. Finally, 18 (37.5%) patients experienced a recurrent weight gain of less than 20% from their nadir weight, yet at the time of GPR had high BMI levels (10 patients had a BMI ≥ 40 kg/m^2^; 7 patients had a BMI between 35 and 39 kg/m^2^) with an enlarged gastric pouch and/or recurrence of comorbidity. One patient was indicated for GPR with a BMI below 30 kg/m^2^ due to the recurrence of a major comorbidity and an endoscopically confirmed wide gastrojejunostomy.

### Intraoperative Results

In 91.7% (44 out of 48) of GPR, a redo of the gastrojejunostomy was performed. Of these, 25% (12 out of 48) were done using a circular-stapled anastomosis (CSA), while 66.7% (32 out of 48) were performed with a linear stapler anastomosis (LSA). Additionally, 8.3% of patients (4 out of 48) underwent resection of a massively enlarged gastric fundus alone without redoing the gastrojejunostomy.

### Weight Progression

**Fig. 2:**
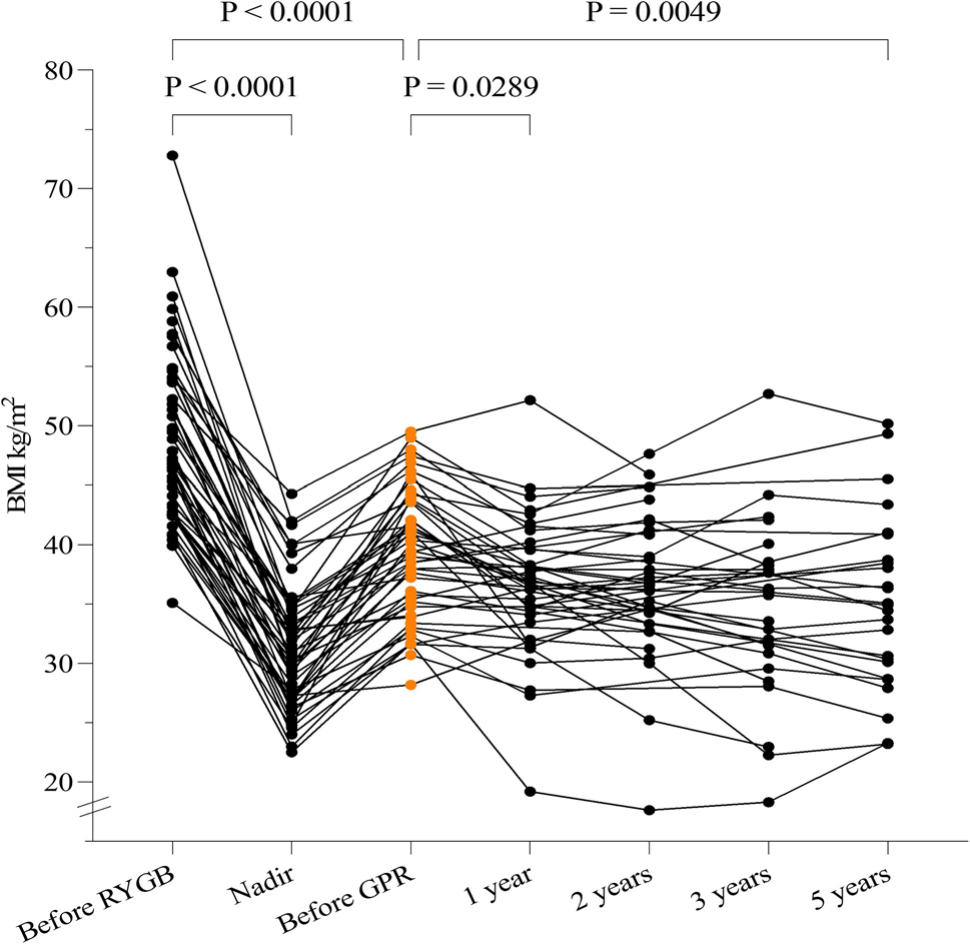
Weight progression over time and treatment

The initial mean BMI before RYGB was 49 ± 7.3 kg/m^2^. A mean nadir-BMI of 31 ± 5.2 kg/m^2^ was reached after 26.3 ± 19.4 months. GPR was performed at a mean BMI of 39 ± 5.4 kg/m^2^. The overall mean follow-up after GPR was 55.9 ± 18 months. Follow-ups of 3 months and 1 and 5 years were available for 47 (97.9%), 41 (85.4%), and 25 (52.1%) patients. The follow-up for weight loss is summarized in Table 2 and Fig. 2. There was no difference in weight course after GPR for patients with a circular- or linear-stapled gastrojejunostomy.

**Table 2:** Follow-up for weight loss

Time of follow-up	Mean BMI (kg/m^2^)	% TWL
3 months (47/48)*	37 ± 4.8	8.7 ± 14.5
1 year (41/48)*	37 ± 5.5	7.7 ± 10.3
2 years (32/48)*	36 ± 6.1	7.8 ± 12.3
3 years (24/48)*	35 ± 7.6	10.1 ± 15.5
5 years (25/48)*	35 ± 7.5	9.1 ± 13.1

^*^Available follow-up in parentheses

### Obesity-Associated Diseases

**Fig. 3:**
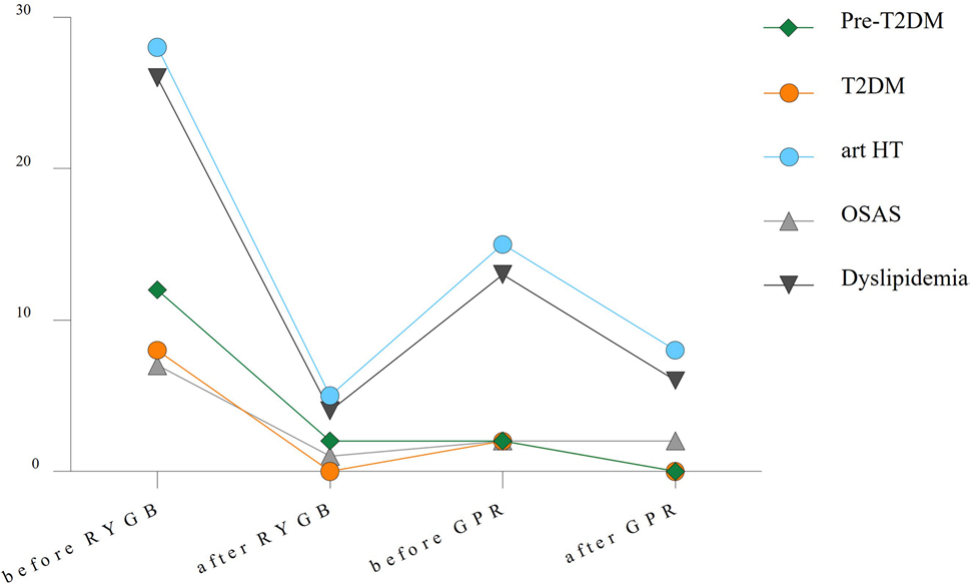
Remission of comorbidities

Information on comorbidities was available for 44 out of 48 (91.7%) patients. Prior to RYGB, *Pre-diabetes* was observed in 12 (25%) patients, decreasing to 2 (4.2%) patients post-RYGB. These 2 patients still suffered from Pre-diabetes at the time of PR. *Diabetes* was present in 8 (16.7%) patients before RYGB with a 100% remission rate thereafter. However, 2 (4.2%) patients experienced a relapse before GPR. At follow-up, none of the patients was still suffering from Pre-diabetes or continuing antidiabetic medication. *Arterial hypertension* was present in 28 (58.3%) patients before and decreased to 5 (10.4%) patients after RYGB. Ten patients experienced a relapse, accounting for 15 (31.3%) patients with arterial hypertension at the time of GPR. At follow-up, 8 (18.2%) patients were continuing antihypertensive medication. OSAS was present in 7 (14.6%) patients before and decreasing to 1 (2.1%) after RYGB. Before GPR, 2 (4.2%) patients were treated for OSAS and were continuing CPAP therapy at follow-up. Dyslipidemia was present in 26 (54.2%) patients before and decreased to 4 (8,3%) after RYGB. Nine patients experienced a relapse, accounting for 13 (27.1%) patients with dyslipidemia at the time of GPR. Six (13.6%) patients were still not within reach of normal lipid values at follow-up. In total, obesity-associated comorbidities present prior to GPR resolved in 52.9% (in 18 of 34, *p* < 0.05) of patients (Fig. 3).

### Postoperative Complications

There was no mortality. Minor complications occurred in 12.5% while major complications occurred in 10.4% of patients within the first year of follow-up.

### In-Hospital Complications

The mean length of hospital stay was 5 ± 1 days. Within that time, three patients presented with a minor complication. One patient suffered from a major complication; an incarcerated incisional hernia was diagnosed prompting surgical revision. The mean CCI at hospital stay was 24.1 ± 5.5 points.

### 90-Day Complications

During the 90 postoperative days, three patients suffered from minor complications. Two major complications were reported. One patient was diagnosed with an anastomotic stricture at gastroenterostomy undergoing dilation therapy. A second patient developed a subcutaneous seroma at the trocar site treated by percutaneous drainage (Table 3). The mean CCI 90 days postoperative was 23.5 ± 4.2 points.

**Table 3:** Early overall complication (90 days)

Patient	Cause	Treatment	CDC	CCI
1	Staple line ulcer	Medical	2	20.9
2	Allergic reaction	Medical	2	20.9
3	Epistaxis	Tamponade	2	20.9
4	Incarcerated incisional hernia	Surgical revision	3b	33.7
5	Reflux disease	Medical	2	20.9
6	Nausea/emesis	Medical	2	20.9
7	Staple line ulcer	Medical	2	20.9
8	Subcutaneous seroma	Percutaneous drainage	3a	26.2
9	Anastomotic stricture	Endoscopic dilation	3a	26.2

*CDC* Clavien-Dindo classification

### Long-Term Complications

The re-operation rate was 8.3% over a mean follow-up of 55.9 ± 18.5 months. Two patients underwent revisional surgery for internal hernias during the first year of follow-up (Table 4). Two other patients received additional secondary bariatric surgery for recurrent weight gain within 5 years after GPR.

**Table 4:** Long-term complications

Patient	Cause	Treatment	CDC
1	Internal hernia	Meso gap closure	3b
2	Internal hernia	Meso gap closure	3b

*CDC* Clavien-Dindo classification

### Patient Satisfaction

**Fig. 4:**
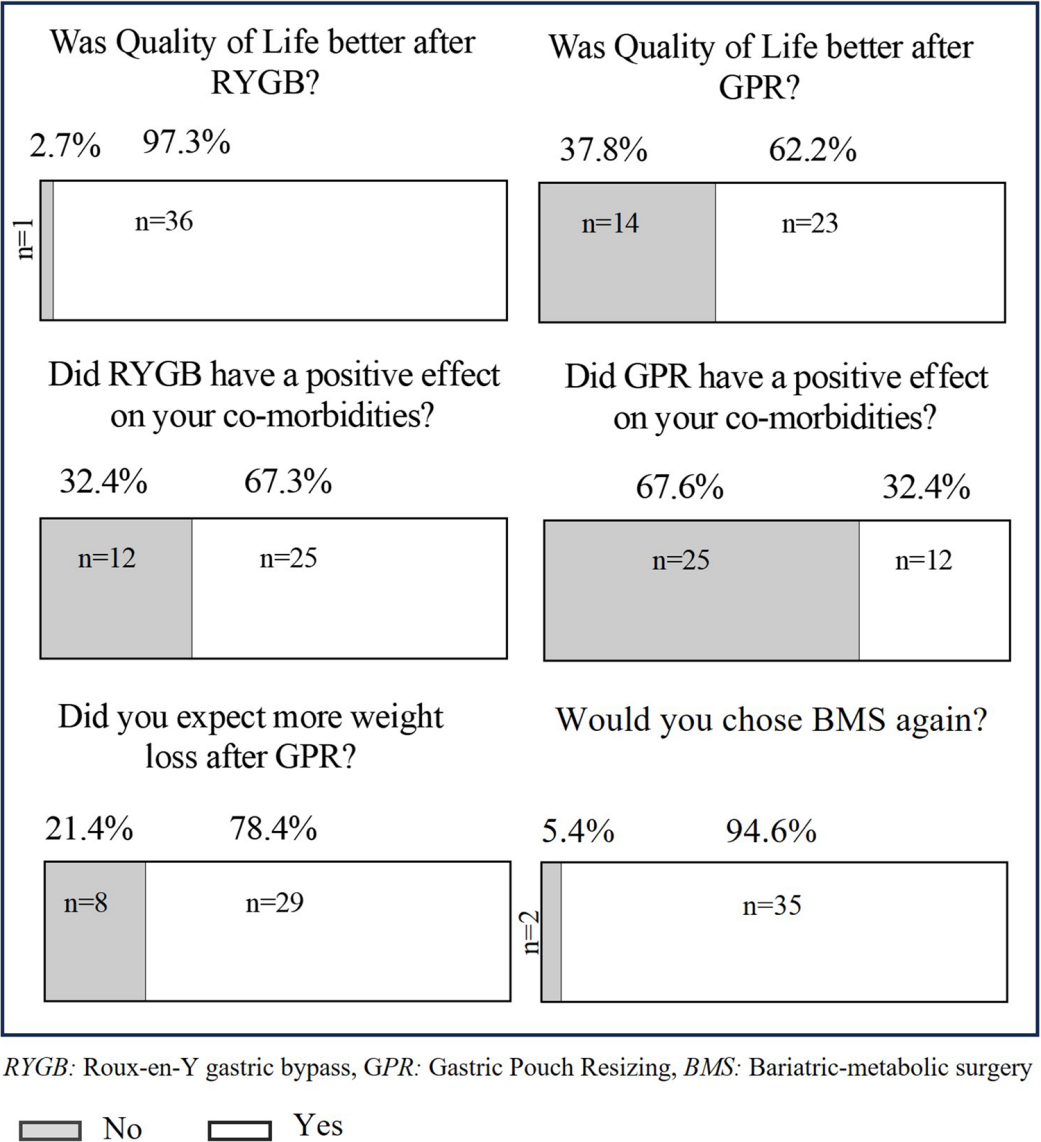
Questionnaire about quality of life

Finally, 37 out of 48 (77.1%) patients participated in our PROM. Ninety-seven percent of patients stated that primary MBS (RYGB) had a positive impact on their quality of life, and 67.2% of patients indicated that primary MBS also positively influenced their comorbidities. A total of 78.4% of patients confirmed that they had expected more weight loss after GPR. Yet, 94.6% of the survey participants would choose both surgeries again (Fig. 4).

## Discussion

Our analysis aimed to understand the effects of gastric pouch resizing (GPR) after RYGB on recurrent weight gain, comorbidities, and patient satisfaction. Key findings include that GPR results not only in weight stabilization but also in a mild weight loss of around 10% over a mean follow-up of 5 years. Second, this technique contributes to the bariatric effect of resolving obesity-associated comorbidities. Finally, GPR is a revisional surgery leading to a high level of patient satisfaction at a low complication rate.

Obesity is a chronic disease turning into a lifelong condition for most persons affected and is associated with comorbidities ranging from wearing effects on the musculoskeletal system to slowly progressing organ failure. The rising prevalence of this chronic ailment places a substantial burden on our healthcare system. Besides, the step-up approach for managing obesity, reaching from conservative to pharmacological and surgical strategies, is not supported by the available evidence and is also far from being spared by therapy failure and relapses requiring further treatment [13]. As is typical for chronic diseases, recurrent weight gain is described in a range from 3.9 to 40% of patients following RYGB [5–7]. Various risk factors and predictors of anatomical, genetic, dietary, psychiatric, and temporal origin have been described [9, 14, 15]. In most cases, it is not possible to identify a singular etiology, which asks for a multimodal instead of uniform treatment. In this manuscript, we focused on enlarged gastric pouches after primary RYGB facilitating recurrent weight gain. The preoperative workup excluded other major reasons for recurrent weight gain, and a multidisciplinary board finally decided on the surgical procedure (in our cases, GPR) accordingly.

GPR is one of the secondary MBS techniques that intend to “adjust” technical failure, other techniques amplify the primary intention of MBS to re-increase weight loss. The former may lead to an improvement of the primary technique (e.g., resizing gastric pouches), and the latter includes techniques that aim to increase restrictive and/or malabsorptive components of primary MBS. Overall, of all secondary MBS, almost 50% are performed to correct recurrent weight gain or suboptimal weight loss [16]. Those amplifying restriction alone after RYGB, focus mainly on the gastric pouch as in GPR. Whether an additional redo of the gastrojejunostomy is improving the result is not entirely clear. According to Wijngaarden et al. comparing GPR alone versus GPR with a redo of gastrojejunostomy, the only difference is the use of surgical disposable, which is lower for GPR alone [17]. GPR is performed for so-called “dilated” gastric pouches. Iannelli et al. introduced the description of a “primary” or “secondary” dilation of the gastric pouch in 2013 [18]. A primary dilated gastric pouch was described as a technical failure during RYGB. The understanding of a secondary dilatation was probably the idea of a repeated overstretching of the gastric pouch. Nowadays the hypothesis of secondary dilated gastric pouches has vanished, due to the fact that all studies that describe gastric pouch dilatation as a potential mechanism for recurrent weight gain after primary RGYB lack a control immediately after surgery to demonstrate that the gastric pouch was initially non-dilated [19–21].

Gastric pouches with a large fundus due to technical failure seem to benefit from a resizing which may lead to weight stabilization or even mild weight loss [18, 21–24]. The optimal clinical response to secondary MBS must be weighed against the risk of the treatment and the possible side effects. In our study with a follow-up of 5 years, weight stabilization was reached by GPR, and—even more important—a resolution of recurrent obesity–associated comorbidities was achieved in most cases.

However, over the last decade, modifications of GPR techniques were reported with the aim of enhancing restriction by adding gastric bands or minimizer rings. A comparison of four different techniques (GPR, gastric pouch banding, GPR and banding, shortening of the common limb) demonstrated no significant difference in terms of additional weight loss [25]. Signs of malnutrition were only seen in patients with a shortened common limb and dysphagia only after applying rings to the gastric pouch, whereas the complication rate for GPR alone was very low. Amor et al. reported their results after GPR for recurrent weight gain in 48 patients and emphasized that the best results were achieved in carefully selected patients: gastric pouch size > 200 ml and/or GJ anastomosis > 20 mm including extensive preoperative evaluation [20].

The challenge lies in finding the best treatment strategy for recurrent weight gain where the benefits outweigh the risks and side effects. Similar to our results, a recent study with a follow-up over 10 years after GPR found that despite recurrent weight gain, the resolution of comorbidities was maintained over time [26]. However, the cohort was rather small including only 20 patients. In a recent meta-analysis, Koh et al. found high remission rates for comorbidities after secondary MBS, especially after revisional duodenal switch [27]. In line with that, Chierici et al. found biliopancreatic diversion with a duodenal switch to guarantee the best results for weight loss, yet at the cost of increased risk for major morbidity [28]. Finally, our study is the first to use PROMs to investigate patient satisfaction and quality of life after secondary MBS. Against the background of a follow-up period of 5 years and a high response rate, we consider these results as representative. Overall, secondary GPR exerted a positive influence on the quality of life, albeit with a lesser impact compared to the improvement that is observed after primary RYGB. This observation can be attributed to an elevated baseline quality of life following primary RYGB [29–31], and the weight loss achieved after secondary MBS typically falls below that attained after initial MBS. Both facts contribute to the surgery’s impact on the patient’s life. Accordingly, over two-thirds of the survey participants expected more weight loss after GPR. It is therefore important that the surgeon manages these expectations. Patients need to know that secondary MBS has a different measure of optimal clinical response than primary MBS. Cessation of recurrent weight gain and resolution of recurrent comorbidities should be considered optimal clinical response. Yet, most survey participants would choose MBS again to treat their obesity, even if they had to undergo several surgeries.

There are limitations to our study. First, we had to deal with heterogenous preoperative workup results, as patients were referred by specialists. Further, both distal and proximal RYGB prior to GPR are included. However, we are confident that this does not confound our findings since distal RYGB is not associated with greater BMI reduction than proximal RYGB [32].

## Conclusion

GPR for recurrent weight gain after primary RYGB is a safe procedure promoting weight stabilization or even mild weight loss as well as a resolution of obesity-associated comorbidities in well-selected patients. The goal in the future needs to be the evaluation of combinational therapies including surgical and pharmacological means based on different stages of the disease as well as timeline regimes while the benefits must outweigh the risk of postoperative complications—hence, personalized surgery with individualized indication appears to be key.

## Supplementary Information

Below is the link to the electronic supplementary material.

Supplementary file1 (JPG 319 KB)

## Data Availability

The data that support the findings of this study are available on request. The data are not publicly available due to privacy/ethical restrictions.
