# Supplementary figures and images for: Gastric Pouch Resizing for Recurrent Weight Gain After Roux-en-Y Gastric Bypass—Does It Have Its Rational?
Source: Obes Surg. 2024 Nov 12;34(12):4369–77. doi: 10.1007/s11695-024-07581-y (PMC11671430; doi:10.1007/s11695-024-07581-y)

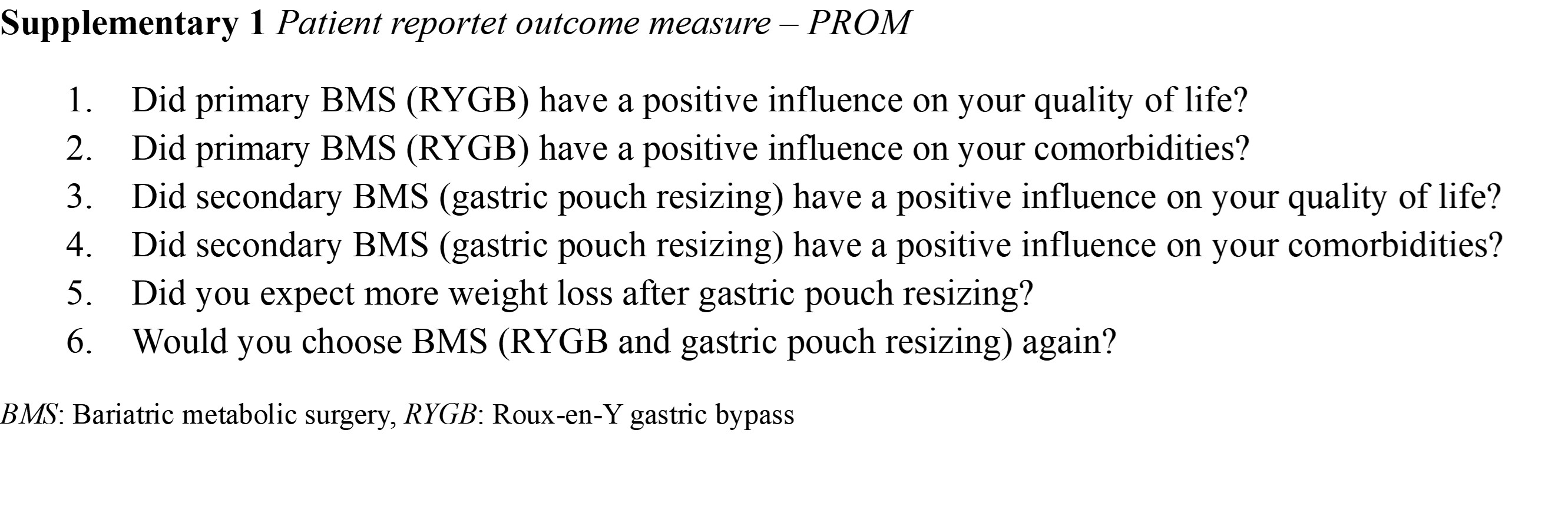

Supplement: Supplementary file 1 — Supplementary file1 (JPG 319 KB) [file 11695_2024_7581_MOESM1_ESM.jpg]
